# Single-cell transcriptomics in planaria: new tools allow new insights into cellular and evolutionary features

**DOI:** 10.1042/BST20210825

**Published:** 2022-10-25

**Authors:** Helena García-Castro, Jordi Solana

**Affiliations:** Department of Biological and Medical Sciences, Oxford Brookes University, Oxford, U.K.

**Keywords:** planarian, regeneration, single-cell transcriptomics, stem cells

## Abstract

Single-cell transcriptomics has revolutionised biology allowing the quantification of gene expression in individual cells. Since each single cell contains cell type specific mRNAs, these techniques enable the classification of cell identities. Therefore, single cell methods have been used to explore the repertoire of cell types (the *single cell atlas*) of different organisms, including freshwater planarians. Nowadays, planarians are one of the most prominent animal models in single cell biology. They have been studied at the single cell level for over a decade using most of the available single cell methodological approaches. These include plate-based methods, such as qPCR, nanodroplet methods and *in situ* barcoding methods. Because of these studies, we now have a very good picture of planarian cell types and their differentiation trajectories. Planarian regenerative properties and other characteristics, such as their developmental plasticity and their capacity to reproduce asexually, ensure that another decade of single cell biology in planarians is yet to come. Here, we review these characteristics, the new biological insights that have been obtained by single-cell transcriptomics and outline the perspectives for the future.

## Introduction

Single-cell transcriptomics is a recently developed technology [1]. However, it has experienced an exponential evolution in the last years [2], with the development of numerous and diverse protocols. In the planarian field, single-cell transcriptomics has been used for more than a decade [3]. Indeed, planarians are one of the most interesting and prolific models for single-cell studies. With this review, we aim to provide a historical and technical overview that help to understand how planarians became a reference model in single-cell transcriptomics, and why they will continue to be in the future.

Planarians have been classical models of stem cell biology and regeneration thanks to their unique characteristics [4]. The regulation and differentiation of stem cells are key questions in biology. However, their study has been traditionally complex. To understand the relevance of planarians in these areas, the limitations of other model organisms have to be noted. Mammalian pluripotent stem cells only exist in the first stages of development, before gastrulation. This makes their study technically difficult and costly, in addition to the ethical implications. Stem cell studies in invertebrate adult animals are also complicated without the use of genetic tools. Well-developed genetic model organisms, like fruit flies or nematodes, lack pluripotent stem cells in their adult forms and show modest or absent regeneration properties.

In contrast, adult planarians possess a large population of stem cells that can be studied during their whole lifespan, called neoblasts [5]. Planarian neoblasts are characterised by the expression of *piwi* [6], *vasa* [7], *bruno* [8,9] and *tudor* [10] genes. These genes have been found to be expressed in the germline of almost every living animal studied [11], but also in many invertebrate pluripotent or multipotent stem cells [12,13]. Neoblasts are small, round cells with a high nucleus/cytoplasm ratio, and are characterised by the presence of chromatoid bodies. These are RNA granules present in the periphery of their nucleus, and also resemble the germ granules present in germ cells of many animals [11]. All planarian cell types differentiate from this unique adult pluripotent stem cells [14]. Planarian neoblasts are constantly self-maintaining and differentiating. Thus, all differentiation stages from pluripotent stem cells to each of the cell types that make the animal, including progenitors, are present in just one sample: the wild type adult. Apart from the normal cell turnover, neoblasts drive planarians’ amazing regenerative capacities. Planarians are able to regenerate all body parts, including the brain, in a matter of days [4]. Besides, planarians are remarkably plastic. Under starvation, they undergo a degrowing process, adjusting their body proportions to their new size [15]. Furthermore, some planarians use their regenerative properties to reproduce asexually by fission, cutting themselves into two or more pieces that will later regenerate into full organisms [16].

These features have made planarians invaluable model organisms for stem cell biology. However, their potential was limited by the difficulty of tracing cell linages in adult individuals and the lack of transgenic tools required to generate modified lines. Fortunately, the introduction of single-cell methods has changed the way we approach the study of cell types and cellular dynamics, opening new horizons for planarian studies [17–21]. In turn, as planarians contain a snapshot of the entire differentiation tree, they have been key for the development of single-cell linage reconstruction algorithms, like PAGA [22].

In this review, we will deepen into the factors that favoured single-cell studies in planarians, like the early development of dissociation and sorting protocols. We will also review the evolution of single-cell methods and how the introduction of increasingly powerful technologies has contributed to the characterisation of planarian neoblasts, differentiated cell types, differentiation trajectories and regeneration processes. In addition, we will present the case of the parenchymal linage, an understudied group of planarian cells that has re-emerged with single-cell sequencing studies, and which origin and functions are still controversial. Finally, we will define the current technical state of single-cell methods, and the perspectives of a future in which the cell atlas era will be overcome in pursuit of more complex single-cell studies.

## Evolution of single cell methods

Single-cell transcriptomics, also called single-cell RNA-sequencing (scRNA-seq), has emerged in recent years as an evolution of bulk RNA-seq. It profiles the expression of individual cells, enabling transcriptomic studies at single-cell resolution. Despite being a recently developed technology, scRNA-seq has experimented a steep expanse in the last decade. The number of cells profiled per experiment has grown exponentially every year, and the technique has gained great popularity among the scientific community [2]. Most scRNA-seq protocols start with the synthesis of cDNAs by poly-A capture of the mRNAs. Other than this, each single-cell approach follows different strategies to isolate the cells and process the cDNA. According to where reverse transcription and cell barcoding take place, we can classify scRNA-seq protocols into three main categories: plate-based, droplet-based, and *in situ* barcoding-based [2,23].

Plate-based protocols capture each cell inside an individual well of a plate (or chip). In these wells, cells are lysed, and RNA is reverse transcribed to cDNA. The first scRNA-seq paper was published in 2009 and reports the profile of a single mouse blastomere, manually isolated in a tube [24]. Later, Smart-seq [25] was the first strategy to capture full-length transcripts using template switching. In 2014, the automatisation of scRNA-seq by liquid handling robots was implemented with MARS-seq [26]. Robotic automatisation substituted manual pipetting for adding reagents and pooling cells, which helped to scale up throughputs from hundreds to a few thousand cells per experiment.

**Figure 1.**
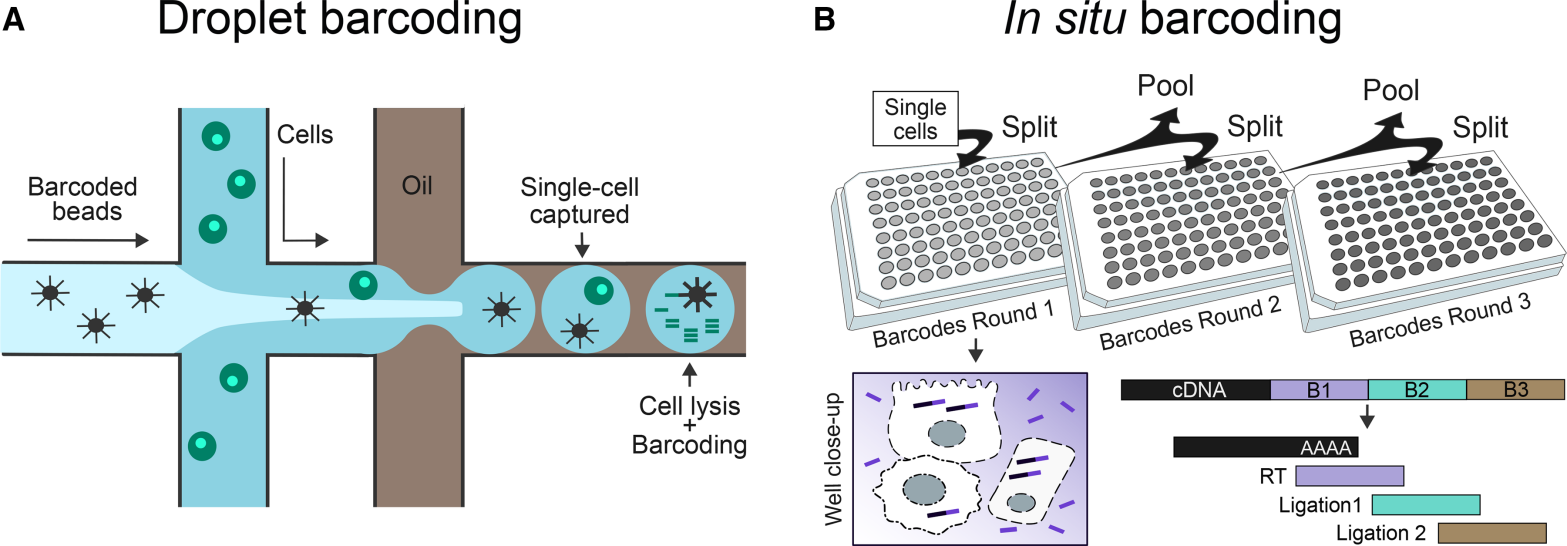
(**A**) Detail of the cell capturing site of a microfluidic device: In Droplet barcoding methods, cells are encapsulated and processed within nanodroplets using microfluidic devices. These have different flow currents for cells (green) and synthetic beads (black). Where these flows merge, a series of nanolitre droplets is created. The flow is adjusted in such a way that each droplet receives a single cell and a barcoded bead, as well as the reagents required for cell lysis and cDNA synthesis. During reverse transcription in the droplet, cDNAs are labelled with unique barcodes carried by the synthetic beads. (**B**) Scheme of split-pool barcoding. In *in situ* barcoding methods, cells are subjected to multiple split-pool rounds. The initial pool of cells is randomly split in a plate, and all the cells falling in the same well are labelled with the same well-specific barcode. Cells are then pooled together and split again in another plate with a different set of barcodes, and the labelling process is repeated. After multiple rounds, each cell is randomly tagged with a unique combination of barcodes, as the probability of two cells falling consecutively into the same wells, and receiving the same barcodes, is minimal. The cDNA is labelled within the permeabilized cell, as seen in the well-close up. Normally, first barcode (purple) is introduced during an indexed reverse transcription, while subsequent barcodes (green and brown) are attached by ligation reactions.

Later established droplet-based protocols are based on microfluidics and were a significant step forward for single-cell transcriptomics. The first methods, InDrop [27] and Drop-seq [28], were developed independently by two different labs in 2015. These protocols use microfluidic devices to isolate cells within a nanodroplet, where they are tagged with a common cell barcode and a unique molecular identifier (UMI) (Figure 1A). The company 10× Genomics popularised this technology with their platform Chromium, which is the current favourite choice for single-cell studies [29].

Finally, *in situ* barcoding is the most recent approach to single-cell transcriptomics and was implemented with the development of SPLiT-seq and sci-RNA-seq [30,31]. In these protocols, the cell itself is used as reaction chamber. This avoids the use of complex cell capturing devices, as cell isolation is no longer required. After tissue dissociation, cells are fixed and permeabilized, so the barcodes and reagents can get through the membrane and reach the mRNAs. Then, reverse transcription and barcoding take place *in situ* within the cell. Cell labelling is based on combinatorial indexing, also known as split-pool barcoding (Figure 1B). A strength of combinatorial indexing is its high scalability. Theoretically, the throughput is only limited by the number of possible barcode combinations. By increasing the number of barcodes or labelling rounds, this approach could profile millions of cells per experiment [32].

## Dissociation approaches in planarians

The first step of single cell transcriptomics is the generation of a single cell suspension. Dissociation techniques in planarians long predated the onset of single cell transcriptomics. The first dissociation approach applied to planarians was non-enzymatic [33], and has been used in microscopy since the 19th and 20th centuries. This approach used an acetic acid-based formula to dissociate tissues preserving the morphology of the cell. Morphology was, in fact, the first approach to classify cell types. Cell dissociation using acetic acid or other acidic formulas has traditionally been known as maceration. It was first used to dissociate Hydra [34,35], and then adapted to planarians by Baguñà and Romero in 1972 [33]. Based on morphological observations, they classified 13 main cell types in two planarian species, and analysed their distribution during growth, degrowth and regeneration. Despite its early use in microscopy, maceration became rare and restricted to a few applications [36,37].

In 2006, Hayashi and co-workers introduced trypsin dissociation in planarians [3,38]. They also implemented a FACS sorting approach comparing the profiles of X-ray irradiated and non-irradiated animals. As a result, planarian cells were isolated and classified in three distinct cell populations: X1 (X-ray sensitive proliferating neoblasts), X2 (X-ray sensitive cell progenitors) and XIS (X-ray insensitive differentiated cells). Since then, enzymatic dissociation protocols based on trypsin, or other enzymes, became widespread for FACS sorting in planarians [39]. The existence of these sample preparation protocols facilitated the subsequent transcriptomic studies, which kept using FACS for cell purification or enrichment, and enzymatic digestion for tissue dissociation [40–42].

Nonetheless, enzymatic cell dissociation is a live process that introduces cell stress [43–45]. Also, live cell dissociation requires of additional treatments, like fixation or cryopreservation, to preserve and storage the cells. To overcome these constrains, non-enzymatic acidic-based formulas were recently rescued to create ACME [46], a cell dissociation-fixation protocol based on the maceration solution [33], with modifications to make it compatible with modern scRNA-seq technologies. ACME fixes the cells while they are being dissociated, preventing the stress of enzymatic digestion, and leaves them ready for freezing or processing in a single protocol.

## Early plate-based single-cell studies reveal planarian neoblast heterogeneity

Single-cell transcriptomic studies in planarian began in 2010, combining enzymatic dissociation and FACS with plate-based reverse transcription PCR (RT-PCR). The first study of this kind analysed gene expression in isolated single-cells from the planarian species *Dugesia japonica* [3], revealing the first insights of planarian stem cell heterogeneity. Later, enzymatic dissociation and FACS, coupled with single cell qPCR, were used in a panel of 96 genes, including many transcription factors [47]. This study managed to classify neoblasts in three different subpopulations: ζ (zeta) neoblasts, which give rise to multiple cell lineages; σ (sigma) neoblasts, which have a broader linage potency and can even regenerate zeta-neoblasts, and γ (gamma) neoblasts, a sub-population contained within sigma neoblasts which was proposed to give rise to the intestine. Previous to these single-cell studies, transplantation experiments in planarians had identified clonogenic neoblasts (cNeoblasts) as a pluripotent population able to differentiate into any cell type [14]. This cNeoblasts were lately suggested to be contained within the sigma population.

All these specialised neoblast subpopulations express different levels of *piwi-1*, the canonical neoblast marker [6], plus a specific set of transcription factors. This fact has been noted by *in situ* hybridisation [48] as well as single cell transcriptomics [17,20,47]. A more detailed exploration of these transcription factors allows to classify neoblasts in even more specialised subtypes [48,49] (e.g. nu-neoblasts has been proposed as neural progenitors [18]. However, neoblast pluripotency, classification and differentiation are still under discussion. For instance, it was recently proposed that every specialised neoblast population may retain pluripotency and act as a cNeoblast under the right circumstances [50].

Single-cell qPCR methods were overcome by Smart-seq, another plate-based method that allowed to increase the number of cells and transcripts profiled per study. In 2015, Smart-seq was applied to enzymatically dissociated FACS-sorted cells to profile the transcriptome of 619 cells, classify them into 13 main cell types, and study gene expression response to injury in different tissues [20]. A further study using the same technique offered detailed insights into planarian epidermal differentiation [21].

## Droplet based methods characterise the planarian cell type atlas

**Figure 2.**
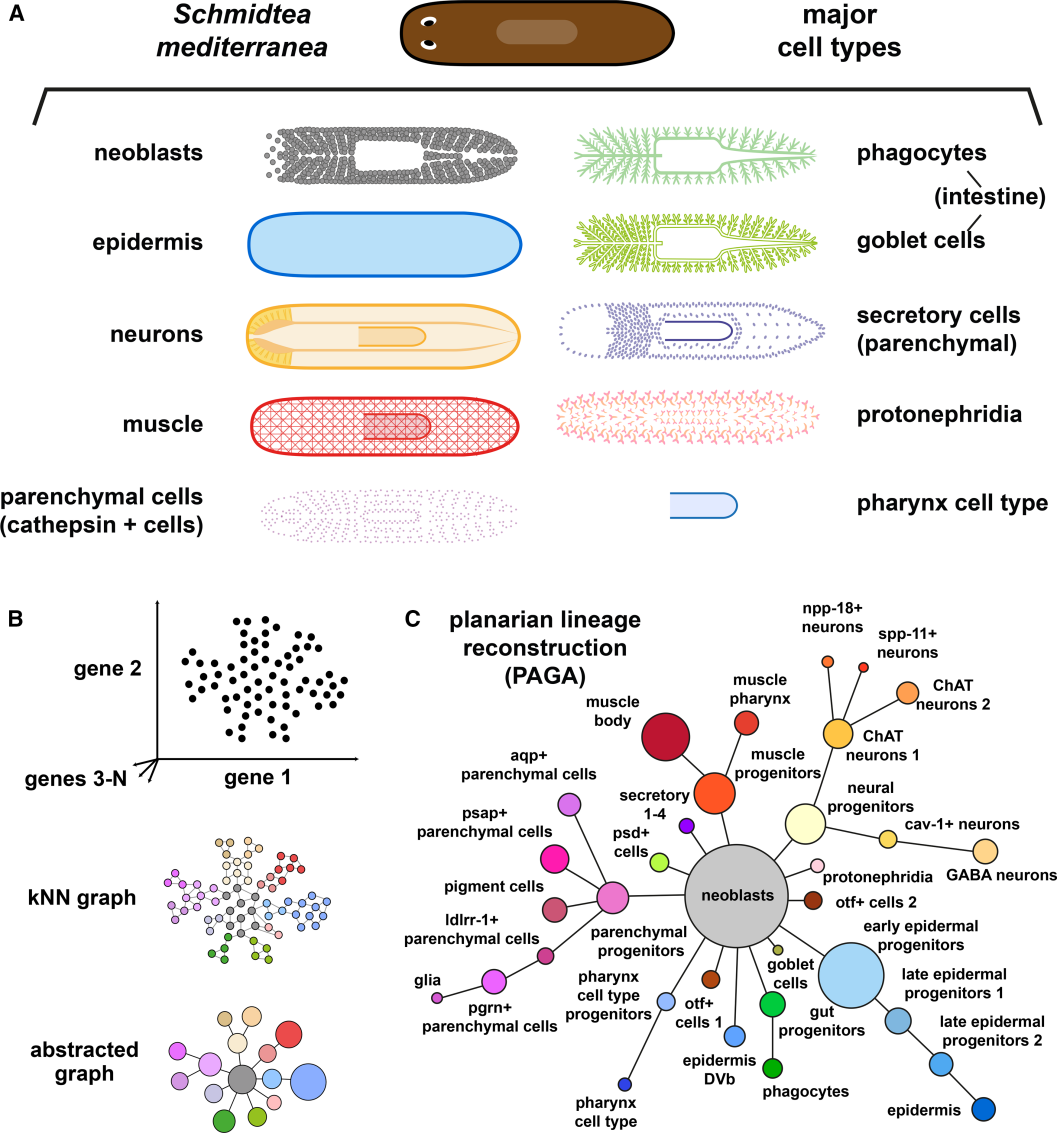
(**A**) Major planarian cell types: epidermis, neurons (including a range of neuron subtypes), muscle (including body and pharynx muscle), parenchymal cell types (also known as cathepsin + cells, including a range of cell types), gut phagocytes, gut goblet cells, secretory cells (including a range of subtypes), protonephridia (including flame and tubule cells), and the pharynx cell type. (**B**) Schematics of lineage reconstruction by PArtition-based Graph Abstraction (PAGA). One way to represent single cell datasets is constructing a kNN graph that connects each cell to its k nearest neighbours in the single cell transcriptomic space. PAGA uses this graph to reconstruct the differentiation lineages. PAGA evaluates the connectivity between different clusters at the kNN graph level. Essentially, the connectivity arises by the existence of cells that are in an intermediate state between both cell clusters, suggesting that cells from one cluster can differentiate into other cell clusters. This reveals the likely connections between clusters and associates a *P*-value to each connection. (**C**) schematisation of the PAGA based planarian lineage reconstruction of the major planarian cell types, based on Plass et al. [19].

With the introduction of droplet-based methods, two comprehensive single-cell atlases of *S. mediterranea* were published simultaneously [17,19]. Both of them using enzymatically dissociated FACS-sorted cells. These publications showed much higher resolution than previous studies, profiling 50 562 and 21 613 cell transcriptomes, respectively, and identifying over 40 cell populations. Planarians atlases were made of a myriad of cell types (Figure 2). As pointed out by early microscopy studies, neoblasts are one of the most abundant (∼30%) populations. Other than stem cells, the major cell types in planarians include epidermis, muscle, and neurons that can be further subclassified in different cell progenitors and fully differentiated populations. Another broadly abundant cell types are the parenchymal cells, also known as *cathepsin+* cells [17], which are of enigmatic origin and function. These studies also identified markers of both phagocytes and goblet cells, the most prominent cell types in the gut. Other less abundant planarian cell types are the secretory and protonephridia cells. Both atlases also identified a cell type specific of the pharynx. Collectively, these single-cell studies presented a wealth of markers from all major cell types of planarians, and allowed the reconstruction of their differentiation trajectories from a single pluripotent cell type.

## *In situ* barcoding methods scale up single-cell studies in planarian

Planarians have been amongst the first organisms to be studied by *in situ* barcoding scRNA-seq methods. Using SPLiT-seq [46], the single-cell atlases of two planarian species (*Schmidtea mediterranea* and *Dugesia japonica*) were profiled together in one single experiment, obtaining results compatible with previous publications [17,19]. The study also confirmed the presence of germ line progenitors, previously described in the literature [51], in both asexual planarian species. On the technical level, one run of SPLiT-seq was able to profile ∼40K cells, the equivalent of running multiple droplet-based experiments. Later, SPLiT-seq was used to profile ∼300K cells of *S. mediterranea* to study transient cellular states in regeneration [52]. The publication identified diverse molecules which expression in muscle, epidermis and intestine contribute to stem cell proliferation and tissue organisation.

## Lineage reconstruction in planarians

Lineage tracing experiments have been key for developmental biology, but cannot be performed in every animal system. Molecular tracers such as dyes excel when injected in embryonic cells, but are difficult to use in smaller cells of adult organisms. Transgenic tracers instead are more versatile, but need well developed transgenic tools, making these techniques unfeasible in the vast majority of animals. Single cell transcriptomic data has been used to develop trajectory reconstruction algorithms as an alternative to these molecular tools [53]. As opposed to lineage ‘tracing', lineage ‘reconstruction' refers to the computational inference of developmental and/or differentiation lineages based on single cell data. Planarian data was key in the development of one of these algorithms, called PAGA [19,22]. PAGA uses graph mathematics to reconstruct the differentiation lineages, revealing the likely connections between clusters and associating a *P*-value to each of these connections. Based on PAGA, the Potency Test was also introduced [19] as another graph-based analysis that quantifies the connectivity of cell clusters. There are also other alternatives for identifying stem cells by combining single-cell transcriptomic data and graph analysis, such as StemID [54]. Here, the number of clusters into which a given cell cluster can differentiate estimates the developmental potency of the cluster.

Linage reconstruction has broadened our understanding of cell type differentiation in planarian and established clear links between different cell types and their progenitors [19]. However, it has also brought surprising insights, like the connection of unexpected cell types with the parenchymal linage, that rise new biological questions.

## The parenchymal cell types

One of the most understudied cell types in planarian are the parenchymal cells, which represent an interesting case for future research. These cells are part of the connective tissue of planarians, known as parenchyma, which also contains neoblasts, progenitors and secretory cells [17,19], among others. Some parenchymal cell populations were characterised by morphology in early microscopy literature, where they are referred to as ‘fixed’ parenchymal cells to distinguish them from ‘free’ mobile neoblasts [33,55,56]. These ‘fixed’ parenchymal cells were described as highly polymorphic, full of vesicles and lipid droplets. After that, parenchymal cell populations have been largely overlooked in molecular studies, with only a handful of publications mentioning them [57–59]. Fortunately, single-cell transcriptomics has re-emerged the parenchymal cells as one of the major cell fates in planarians [17,19,46], and revealed their diversity through the identification of new cell identities.

Single-cell transcriptomic studies have named these identities according to the expression of characteristic gene markers. Thus, some studies refer to the whole parenchymal lineage as *cathepsin+* cells [17], while others classify each cell type according to a distinct marker [19,46]. Here, we will use the latest nomenclature. According to lineage reconstruction experiments based on single-cell data, the parenchymal lineage contains parenchymal progenitors, which give rise to all other identities. Among them are *aqp+* cells (aquaporin positive), *psap+* cells (prosaposin), *pgrn+* cells (progranulin) and *ldlrr-1+* cells (low density lipoprotein receptor-related 1). These groups are likely to correspond to the ´fixed´ parenchymal cells previously described by microscopy. Additionally, single-cell studies indicate the parenchymal cell lineage also contains planarian glia and pigment cells [59–62].

Some parenchymal populations are phagocytic [63] and rich in lysosomes, hydrolytic enzymes, and vacuoles. As planarians lack a circulatory system to transport nutrients, these cells may intervene in the distribution and storage of gut metabolites and in the transport of excretory products to the protonephridia. However, these roles remain poorly understood. Parenchymal cells are highly affected by regeneration, size, and starvation [33]. Regeneration decreases the numbers of certain types, like *aqp+* [19]. On the contrary, their numbers increase in bigger and well-feed animals, which indicates parenchymal cells are used as metabolic reservoirs [33]. The parenchymal cells are also intimately connected to planarian stem cells [55], as phenotypes associated to neoblast loss have been reported for genes expressed in parenchymal identities [59,64]. Thus, parenchymal cells are likely to play an important role in planarian regeneration.

On the other hand, the identification of the glia and pigment cells as part of the parenchymal linage by independent single-cell studies poses a conundrum in evolutionary biology. Both glia and pigment cells have an ectodermic origin in vertebrates [65–67]. On the contrary, parenchymal cells may be linked to endodermal tissues, as indicated by the expression of transcription factors like *hnf4*. Planarian glial cells are found entwined to neural cells in the neuropil, and near to brain branches, photoreceptors (eyes) and the peripheral nervous system. They have also been shown to carry out typical glial cell functions [59,62], and one of their proposed roles is to metabolise the excess of neurotransmitters. Interestingly, classic microscopy already associated the morphology of some parenchymal cells with the mammalian glia [56].

The parenchymal origin of planarian glia and pigment cells suggests a disparate evolution of these cell types that have yet to be elucidated. Future comparative studies in planarians and other animal groups will shed light on the evolution of these cell types. In the same way, further research will be required to fully characterise and understand the role of other parenchymal cell types.

## Current state in single-cell transcriptomics and future perspectives for planarian studies

Single-cell transcriptomics is living in the cell type atlas era. Planarian atlases have been profiled by different techniques and authors [17–20]. In the same way, single-cell methods have been used to generate cell type atlases of multiple animal groups, including sponges [68], cnidarians [69,70], nematodes [30,71], arthropods [72], amphibians [73], fish [74] or mammals [75], among many others. These atlases have been useful to identify novel cell types and study cell differentiation trajectories, developmental stages and gene expression at tissue resolution. But in the future, the atlas era will be surpassed by more complex and quantitative studies comparing single-cell datasets across stages, knockdowns, replicates, conditions and species.

This leap is a challenge in several aspects. At the tissue dissociation level, we are constrained by the limitations of classical enzymatic digestion. However, recently developed protocols, like single-nuclei isolation [76,77] or ACME [46], are alleviating traditional sample preparation constrains and allowing more experimental flexibility to collect and store samples. At the single-cell platform level, most studies nowadays use droplet-based approaches [2]. Nonetheless, droplet-based strategies have the disadvantage of limiting the number of cells and conditions per experiment, and of requiring high budgets and specialised equipment. *In situ* barcoding methods can overcome these constrains, and present multiple advantages for the future of single cell transcriptomics. They can be easily scaled up to process hundreds of thousand cells (depending on the configuration of the experiment) in a cost-efficient way, as demonstrated by recent publications in other model organisms [30–32,78]. *In* situ barcoding methods also allow multiplexing. Several samples and conditions can run in parallel in the same experiment, avoiding batch effects. Finally, single-cell transcriptomics is moving towards multi-species comparisons at tissue resolution [79–82].

In the future, these advantages will open the door to complex functional genomics comparisons at cell type resolution in planarians. It will be possible to simultaneously profile several RNAi conditions, including replicates. The resolution to identify novel cell types will be increased by the growing number of cells profiled per experiment. And multi-species comparisons will lead to novel evolutionary insights and help to elucidate questions like the developmental origin of planarian parenchymal cell types.

## Perspectives

- Single cell transcriptomics has allowed to build the single cell atlas of different planarians species, discover novel cell types and reconstruct cell differentiation trajectories.
- This has revealed new biological insights. Single cell studies have enabled to characterise the heterogeneity of the neoblast stem cell population and to gain knowledge of understudied cell types such as the parenchymal cells.
- Future studies will go beyond the cell type atlas era and will use most novel technologies to enquire how planarian cell populations respond to different treatments and conditions.

## Abbreviations

PAGA: PArtition-based Graph Abstraction
scRNA-seq: single-cell RNA-sequencing
